# Room-temperature acetone gas sensing using Sm-doped Co–Zn ferrite nanoparticles: role of mesoporosity and oxygen vacancies in enhancing sensor response

**DOI:** 10.1039/d5na00631g

**Published:** 2025-09-15

**Authors:** Anil B. Mugutkar, Shyam K. Gore, Siddheshwar D. Raut, Sunil M. Patange, Rajaram S. Mane, Shoyebmohamad F. Shaikh, Sagar E. Shirsath, Santosh S. Jadhav

**Affiliations:** a Bahirji Smarak Mahavidyalaya Basmath Hingoli Maharashtra 431502 India; b D. S. M's Arts, Commerce and Science College Jintur Parbhani Maharashtra 431509 India santosh.jadhav28@yahoo.com; c Sharadchandra Arts, Commerce and Science College Naigaon Nanded Maharashtra 431709 India; d Shri Krishna Mahavidyalaya Gunjoti Osmanabad Maharashtra 413606 India; e Swami Ramanand Teerth Marathwada University Nanded Maharashtra 431606 India; f Department of Chemistry, College of Science, King Saud University P. O. Box 2455 Riyadh 11451 Saudi Arabia sshaikh1@ksu.edu.sa; g Department of Physics, Vivekanand College Chhatrapati Sambhajinagar Maharashtra 431001 India; h School of Materials Science and Engineering, University of New South Wales Sydney NSW 2052 Australia s.shirsath@unsw.edu.au shirsathsagar@hotmail.com

## Abstract

This study presents the synthesis, structural characterization, and acetone gas sensing behavior of nanocrystalline Co–Zn–Sm ferrites (CZSmF) with the general formula Co_0.7_Zn_0.3_Sm_*x*_Fe_2−*x*_O_4_ (*x* = 0–0.04), synthesized *via* the sol–gel auto-combustion method. Rietveld-refined X-ray diffraction (XRD) analysis confirms the formation of a single-phase spinel structure (space group *Fd*3̄*m*), with crystallite sizes ranging between 31 and 43 nm as determined by the Williamson–Hall method. Transmission electron microscopy (TEM) and selected area electron diffraction (SAED) reveal well-defined nanocrystals with predominantly oval morphology and an average particle size of ∼28 nm. Brunauer–Emmett–Teller (BET) analysis indicates mesoporous behavior with surface areas ranging from 6.2 to 15.2 m^2^ g^−1^ and pore sizes in the range of 16–22 nm. X-ray photoelectron spectroscopy (XPS) confirms the oxidation states of Co^2+^, Zn^2+^, Fe^2+^/Fe^3+^, and Sm^3+^ ions and reveals significant oxygen vacancies contributing to the gas sensing mechanism. The CZSmF sample with *x* = 0.01 (CZSmF01) demonstrates superior acetone sensing performance at ambient temperature (296 K), exhibiting high selectivity, a swift response time of 84 s, and a rapid recovery time of 24 s for 100 ppm acetone. The optimized sensing performance is attributed to a synergistic combination of favorable crystallite size, pore architecture, and oxygen vacancy-induced n-type conduction. Additionally, CZSmF01 shows high stability and reproducibility across multiple cycles and maintains linear response characteristics across a concentration range of 25–100 ppm, establishing its applicability for quantitative detection. Notably, the sensor also demonstrates humidity sensing with low response (10 s) and recovery (8 s) times, indicating multifunctionality. The results highlight the potential of CZSmF01 for commercial acetone detection applications.

## Abstract 2

The study presents the synthesis, structural characterization, and acetone gas sensing by nanocrystalline Co–Zn–Sm ferrites (CZSmF) with the general formula Co_0.7_Zn_0.3_Sm_*x*_Fe_2−*x*_O_4_ (*x* = 0–0.04), synthesized *via* the sol–gel auto-combustion method.

## Graphical abstract

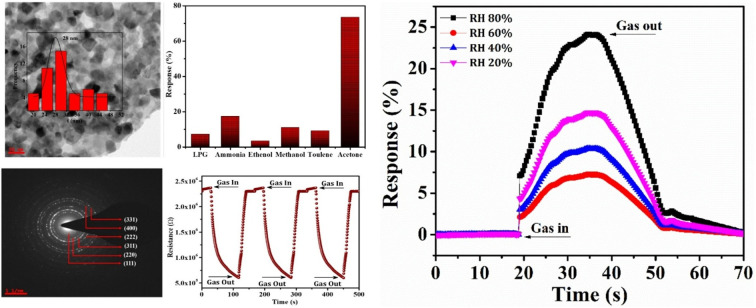

## 1 Introduction

Nanocrystalline magnetic spinel ferrites have gained significant attention in gas sensing applications due to their remarkable chemiresistive properties, swift response/recovery characteristics, long-term stability, and high sensitivity towards a variety of gases.^1–3^ Specifically, the structural versatility and electronic properties of spinel ferrites facilitate the tailoring of their sensing performance through appropriate cationic substitutions and modifications of synthesis methods.^4–6^ Among various spinel ferrites, cobalt ferrite (CoFe_2_O_4_) is particularly promising due to its unique inverse spinel structure, characterized by high coercivity, high Curie temperature, moderate saturation magnetization, and notable electrical properties.^7,8^

Incorporation of Zn^2+^ ions into magnetic cobalt ferrite significantly influences cation distribution, particle size, and spin orientation, thereby modifying its physical and chemical properties for targeted applications. Zn-substituted cobalt ferrites have been extensively studied for their superior sensing performance towards various gases, including ethanol,^9^ liquefied petroleum gas (LPG),^10^ and hydrogen (H_2_).^11^ For instance, Co–Zn ferrite thick films demonstrated considerable sensitivity towards LPG, along with rapid response and recovery times, highlighting their practical applicability.^10^ Moreover, the deposition of palladium on Co–Zn ferrite nanoparticles has further improved their hydrogen gas sensing capability, illustrating the potential for enhancing sensitivity and selectivity through surface functionalization.^11^

Recently, doping rare-earth (RE) ions, such as Gd^3+^, La^3+^, and Sm^3+^, into spinel ferrites has emerged as a novel approach to further enhance their magnetic and gas sensing capabilities by modifying their structural and electronic properties.^12–15^ Sm^3+^ doping, in particular, has shown promising results by significantly altering the crystal structure, reducing crystallite sizes, and increasing lattice strain. These modifications often result in improved sensitivity, faster response, and shorter recovery times for detecting gases like chlorine (Cl_2_), ethanol (C_2_H_5_OH), and LPG.^14,15^

Acetone, an organic volatile compound with high inflammability, poses health risks upon inhalation, necessitating effective sensing mechanisms for its detection at trace levels.^16^ Spinel ferrites such as ZnFe_2_O_4_,^17^ Ce-doped CoFe_2_O_4_,^18^ and NiFe_2_O_4_ (ref. 19) have demonstrated commendable acetone sensing performance, further emphasizing the importance of structural and compositional optimization in gas sensor development. Specifically, rare-earth doping has shown the potential to enhance acetone sensing through the modulation of crystallinity, porosity, and surface chemistry, which directly influences the interaction between gas molecules and the sensor surface.^12,14,15^

Building on prior studies, the present work focuses on synthesizing nanocrystalline Sm^3+^-doped Co–Zn ferrites (CZSmF) *via* a sol–gel auto-combustion route and systematically evaluating their structure, morphology, and acetone sensing at ambient temperature. The novelty of our approach is a catalyst-free sensing strategy that co-designs defect chemistry and mesoscale architecture within a single-phase spinel: rare-earth (Sm^3+^) substitution is leveraged to tune oxygen-vacancy density, lattice strain, cation distribution, and grain-boundary potential barriers, thereby modulating Fe^2+^/Fe^3+^ small-polaron hopping while a mesoporous network is tailored for Knudsen-limited diffusion. Rather than relying on noble-metal sensitizers, heterojunctions, or thermal activation, this integrated “diffusion-reaction-transport” framework reconciles the usual trade-off between porosity and electronic conduction by jointly optimizing the pore-size window and baseline resistivity to maximize surface redox utilization at room temperature. Substituting larger Sm^3+^ for Fe^3+^ is thus anticipated to reshape the structural and electronic environment in a mechanistically guided manner, providing a stable, low-power platform for selective VOC detection. Accordingly, this study aims to elucidate the underlying structure–property relationships in Sm-doped Co–Zn ferrites and establish their suitability as efficient, selective acetone gas sensors.

## 2 Experiment and characterization

### 2.1 Materials and synthesis

The materials used for the sol–gel synthesis of Co_0.7_Zn_0.3_Sm_*x*_Fe_2−*x*_O_4_ (CZSmF) with *x* = 0–0.04 are the analytical reagent grade (99.9%) pure metal nitrates of iron, samarium, zinc and cobalt possessing chemical formulae Fe(NO_3_)_3_·9H_2_O, Sm(NO_3_)_3_·6H_2_O, Zn(NO_3_)_2_·6H_2_O, and Co(NO_3_)_2_·6H_2_O respectively. The fuel utilized for the combustion is citric acid, C_6_H_8_O_7_·H_2_O. The ammonia solution, NH_4_OH, was used to maintain the pH of the solution. The chemicals are provided by Acros and Loba Chemie, India. The chemicals were weighed according to stoichiometric calculations where the metal nitrate to citrate ratio (molar) was kept at 1 : 3. The details of the synthesis technique are discussed in previous research work.^12^ The as prepared powders of the ferrites were sintered in the air atmosphere in a furnace at a constant temperature of 450 °C for 4 h. The electrical and gas sensing studies were performed by using the pellets of the sintered powders. The pellets were prepared by mixing the powder with the binding agent, poly vinyl alcohol (PVA) and keeping the mixture in a stainless steel dye under a pressure of 2.5 tons in a KBr press. The pellets were then sintered at 450 °C for 2 h to make them free from the binding agent, PVA.

### 2.2 Characterization and property measurements

The X-ray diffractograms were recorded on a benchtop model II powder X-ray diffractometer made by Rigaku at room temperature using a CuKα source by maintaining the scanning rate at 2° per minute within the 2*θ* range of 10–80°. The morphological and compositional details were studied from the digital images of the powders recorded on the scanning electron microscope model S-4800 of Hitachi. The transmission electron microscope model G2 S twin of Tecnai was used to record the TEM, HRTEM and SAED images of the selected sample. The surface area and porous structure of the ferrite samples were analyzed from the nitrogen gas isotherms recorded on model v5.2 of Quantachrome. The experiments were performed for the adsorption and desorption of the gas by changing the pressure. The presence of elements along with their valence states in the selected ferrite sample was confirmed from the X-ray photoelectron spectra recorded using the model ESCALAB Xi^+^ of Thermo Fisher Sci. The impedance analyzer model Hioki IM 3570 was used to record the electrical parameters of the ferrites in the form of pellets. The electrical capacity (*C*) and quality factor (*Q*) are the parameters recorded by varying the frequency (*f*) of the electrical field. The alternating current (ac) conductivity (*σ*_ac_) of the ferrite is calculated by using the following formula reported in the literature.^20^

1 *σ*_ac_ = *ωε*_0_*ε*′

where *ε*′ is the dielectric constant of the ferrite given by,

2 *ε*′ = *cb*/*aε*_0_

where *b* is the breadth between the two circular flat surfaces of the pellet, *a* is the area of the surface, *ε*_0_ is the permittivity of free space, and *ω* = 2π*f* is the angular frequency of the electrical field.

### 2.3 Gas sensing measurements

Gas sensing by the ferrites was evaluated by measuring their resistances in air (*R*_a_) and in the gas (*R*_g_) atmosphere. The two-probe method was used for the measurement of the resistance of the pellet using a Keithley 2400 Source Meter. Two silver contacts were applied onto the top surface of the ferrite pellet using a silver paste to ensure surface conductivity. The pellet was inserted into the sensing chamber, which had a capacity of 250 mL. Acetone was then introduced into the glass chamber *via* fine-needle injection, with the desired concentration of the gas measured using a micropipette. After a 15-minute cycle, acetone was released by removing the piston of the chamber. The sensor response was calculated by using the following relation:^21^3
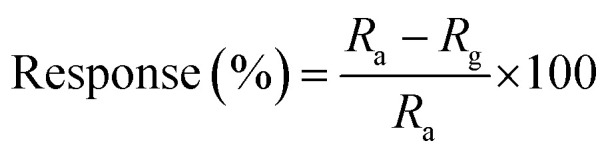
where ‘*R*_a_’ is the resistance of the sensor in the presence of air, and ‘*R*_g_’ is the resistance of the sensor in the presence of acetone gas.

The desired concentration of acetone gas was obtained by the static liquid gas distribution method, and was calculated using the following formula:4
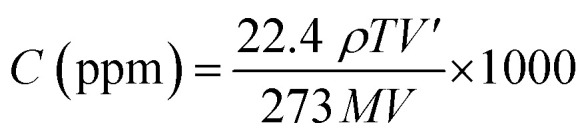
where *C* is the acetone gas concentration (ppm); *ρ* is the density of the acetone liquid (g mL^−1^); *V*′ is the volume of the acetone gas (μL), *T* is the testing temperature (K), *M* is the molecular weight of acetone (g mol^−1^), and *V* is the volume of the testing chamber (L). In this work, the values of *M*, *ρ*, *V* and *T* were 58.08 g mol^−1^, 0.7857 g cm^−3^, 0.250 L and 296 K, respectively.

The sensing of humidity by the sensor was studied by utilizing a binary electrode for the controlled levels of relative humidity varying from 20 to 80%. The relative humidity (RH) level at room temperature was controlled by using saturated salt solutions.

## 3 Results and discussion

### 3.1 Structural characterization

**Fig. 1:**
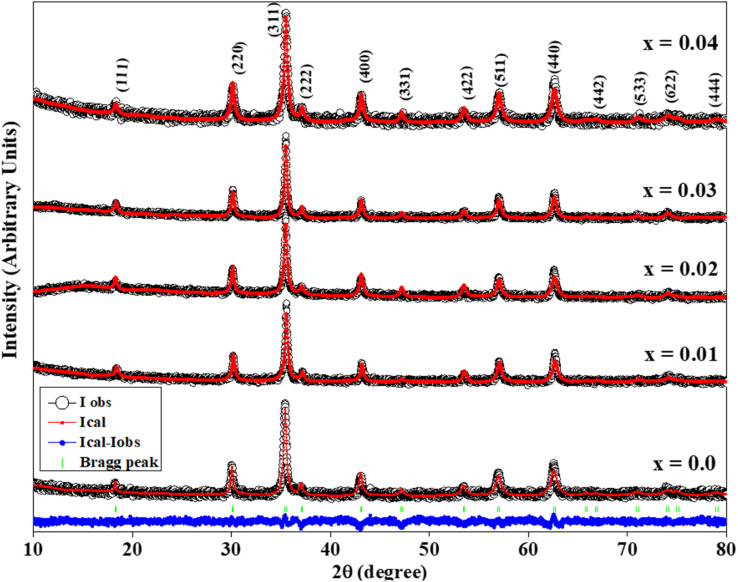
Rietveld refined XRD patterns of Co_0.7_Zn_0.3_Sm_*x*_Fe_2−*x*_O_4_ (CZSmF) ferrites (*x* = 0.00–0.04), confirming the formation of a single-phase spinel structure (space group *Fd*3̄*m*) without impurity peaks. All prominent reflections are indexed and correspond to standard spinel ferrite planes, indicating phase purity and crystallographic stability upon Sm^3+^ substitution.

**Fig. 2:**
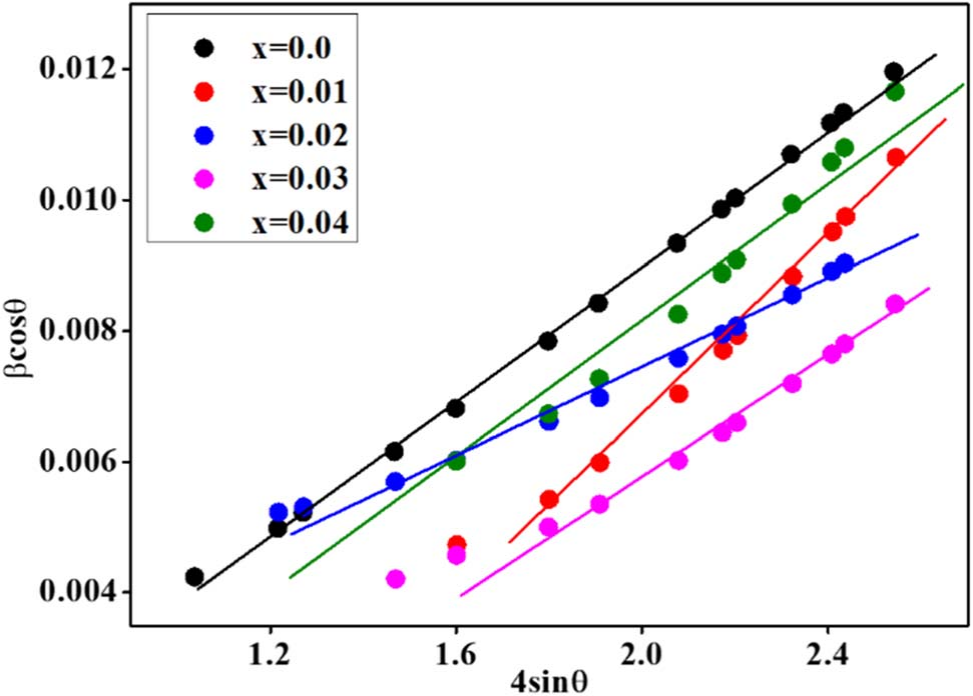
Williamson–Hall (W–H) plots for CZSmF ferrite compositions (*x* = 0.00–0.04). The linear fittings reveal microstrain (*ε*) and crystallite size (*t*) contributions to peak broadening, showing strain-induced lattice distortion associated with Sm^3+^ incorporation due to ionic radius mismatch with Fe^3+^.

The ferrites were structurally characterized from the Rietveld refined X-ray diffractograms (XRD) of the CZSmF, which are displayed in Fig. 1. The Miller indices of the planes point to the corresponding peaks of a typical ferrite sample with composition, *x* = 0.4. The indexing of peaks reveals the formation of a spinel ferrite phase having space group *Fd*3̄*m* within the synthesized CZSmF.^22,23^ The XRD exhibits the formation of Sm substituted Co–Zn ferrites without any impurity since no alien peak corresponding to the phase other than the spinel phase is found in the XRD of the ferrites. This is attributed to the fact that sol–gel auto-combustion is the most suitable method reported for the rare earth ion-substituted spinel ferrites with a single spinel phase.^24,25^ The crystallite size and lattice strain of the CZSmF are obtained from the Williamson–Hall (W–H) method. The W–H plots are displayed in Fig. 2 and the obtained values of crystallite sizes (*t*) and the lattice strains (*ε*) are listed in Table 1. The table exhibits an overall decrease in the values of ‘*t*’ except for the CZSmF with *x* = 0.02 and 0.03. However, the monotonic increase in the values of ‘*ε*’ points towards the continuous decrease in ‘*t*’, since it is reported that for rare earth (RE) doped spinel ferrites, there is an inverse relation between the crystallite size and lattice strain.^26^ The linear increase in the lattice strain with increasing RE composition is due to the larger ionic radius of the doped RE ion *i.e.*, Sm^3+^ (0.96 Å) as compared to the replaced Fe^3+^ ions (0.67 Å).^27^ The decrease in ‘*t*’ is consistent with the fact that, in RE-doped spinel ferrites, the crystallite growth is inhibited by the doped RE elements.^28^ However, the non-linear variation of ‘*t*’ with a linear increase in ‘*ε*’ for the present CZSmF is due to the coalescence of the particles for the ferrites with *x* = 0.02–0.03 during the process of annealing. In ferrites, it is reported that the crystallite size is influenced by the coalescence of the particles during the process of annealing.^12,29^

**Table 1:** The structural parameters of CZSmF obtained from XRD and BET

*x*	*t* (nm)	*ε* (10^−3^)	*a* (Å)	*V*′ (Å^3^)	*A* (m^2^ g^−1^)	*V* (cc g^−1^)	*D* (nm)
0.0	42	3.17	8.399	592.53	—	—	—
0.01	32	3.94	8.411	594.92	6.2116	0.069291	22.31
0.02	33	5.20	8.424	597.83	8.3070	0.095598	23.02
0.03	43	6.32	8.401	592.98	9.0527	0.091280	20.17
0.04	31	6.50	8.392	590.97	15.2336	0.124121	16.30

The lattice parameter (*a*) and unit cell volumes (*V*′) of the CZSmF are calculated by using the formulae reported in the literature^12^ and the obtained values are listed in Table 1. The lattice parameter values increase with the Sm^3+^ composition till *x* = 0.02 and for *x* > 0.02, the values decrease with increasing composition. The increase in lattice parameter values is due to the replacement of smaller Fe^3+^ cations by the bigger Sm^3+^ cations. However, for the larger composition, *x* > 0.02, the decrease in the lattice parameter values points to the presence of lattice imperfections owing to the large difference in the ionic radii of substituted Sm^3+^ and replaced Fe^3+^ ions. The non-linear variation in lattice parameter values due to the bigger size of substituted Sm^3+^ ions is reported in the literature.^30,31^ The compositional variation of the unit cell volume is in accordance with the lattice parameter.

### 3.2 Morphology and compositional analysis

**Fig. 3:**
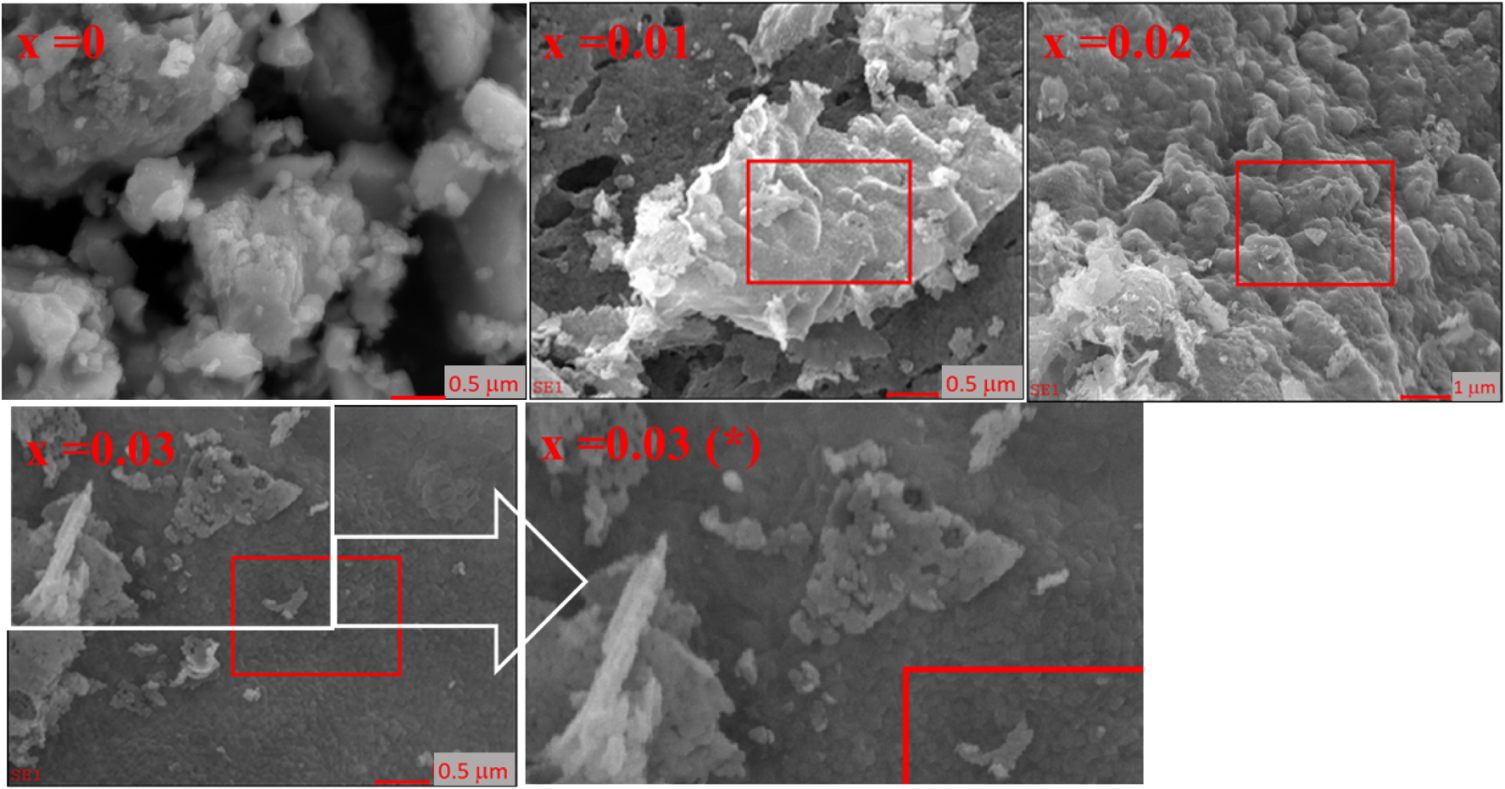
SEM micrographs of CZSmF samples for *x* = 0.00, 0.01, 0.02, and 0.03 illustrating morphological evolution with Sm^3+^ doping. Observed features include spherical to flake-like agglomerates, pore distribution, and particle compaction, indicating textural modifications with increased Sm content. Inset image (*) shows magnified porosity of *x* = 0.03.

The morphology of CZSmF was analyzed from the SEM images (Fig. 3) of the typical samples with *x* = 0, 0.01, 0.02, and 0.03. For the *x* = 0 sample, particle coalescence forming larger grains is evident. The particles in CZSmF with *x* = 0.01 are densely arranged with clearly visible pores larger than the particles. The SEM image for CZSmF with *x* = 0.02 shows particles resembling ash flakes. Particles larger than those in the ferrite with *x* = 0.01, exhibiting fewer pores, are visible. The highly dense structure of fine particles stacked like those in the ferrite with *x* = 0.01 is visible in the SEM image of the ferrite with *x* = 0.03. The selected part of the image of the ferrite with *x* = 0.03 is zoomed in as image *x* = 0.03 (*), for better visualization of the pores and particles. A few pores, larger than the particles, are also visible in the image.

**Fig. 4:**
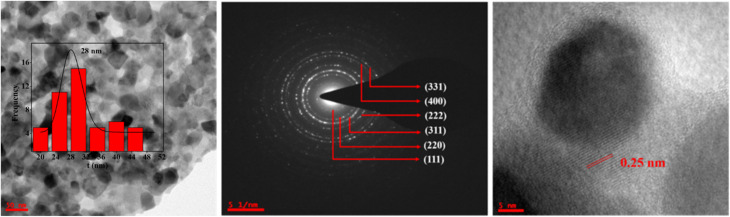
TEM, SAED, and HRTEM analyses of CZSmF01 (*x* = 0.01) confirming nanoscale morphology and crystallinity. TEM reveals predominantly oval nanoparticles with an average size of ∼28 nm. SAED pattern shows clear ring diffraction matching XRD planes, indicating the polycrystalline spinel phase. HRTEM exhibits lattice fringes with a *d*-spacing of ∼0.25 nm, corresponding to the (311) plane.

The shapes and sizes of the CZSmF nanoparticles were confirmed from the TEM profiles. The TEM image of a typical CZSmF with *x* = 0.01 is displayed in Fig. 4. Most of the particles are oval-shaped, while a few exhibit cubic shapes with sharp edges. The sizes of the particles are measured by using the ImageJ software. The histogram of the particle size is embedded in the TEM image. The curve of the statistical fit exhibits a narrow peak at 28 nm, pointing to the average particle size. The crystallinity and phase of the synthesized ferrites are confirmed by recording the selected area electron diffraction (SAED) and High-Resolution TEM (HRTEM) images of the typical CZSmF with *x* = 0.01 (Fig. 4). The spotty diffraction rings observed in the SAED pattern point towards the polycrystalline nature of the CZSmF powders. The computed radii of the rings confirm the peaks revealed in the XRD with the same sequence, thus confirming the spinel phase within the ferrite with the *Fd*3̄*m* space group. The HRTEM image of CZSmF with *x* = 0.01 displays uniformly spaced fringes. The width of the fringe is 0.25 nm, which matches well with 0.252 nm for the (311) peak in XRD and thus confirms the formation of the ferrite phase.

The compositional analysis of CZSmF is performed by recording energy dispersive X-ray spectra (EDS) for the Sm^3+^ doping concentrations *x* = 0.01–0.04. The obtained spectra are displayed in Fig. S1 of SI 1. The pie charts drawn from the atomic weight percentages of the elements show that the CZSmF samples are composed of their constituent elements in accordance with the expected stoichiometry, where the ratio of (Co + Zn) : (Fe + Sm) is maintained at 1 : 2.

### 3.3 Porous structure and surface area

**Fig. 5:**
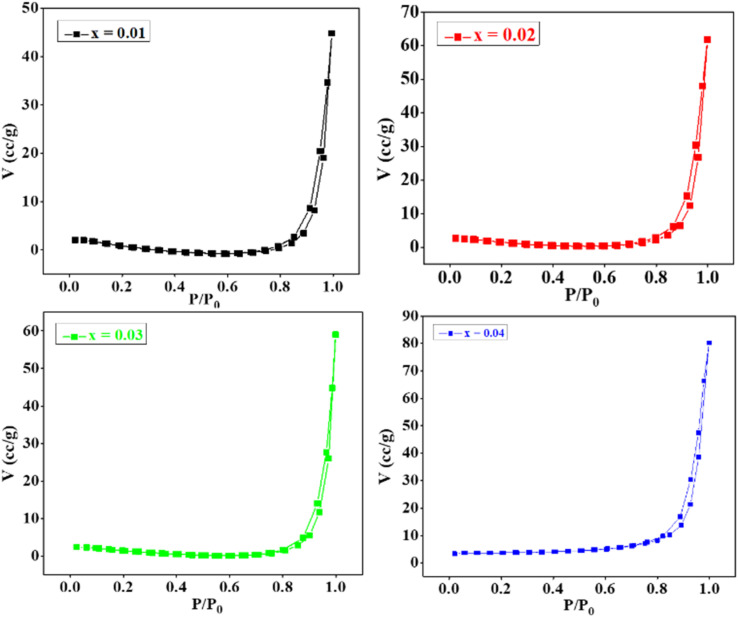
BET N_2_ adsorption–desorption isotherms for CZSmF ferrites (*x* = 0.01–0.04) showing H3 hysteresis loops, characteristic of mesoporous materials. Increased Sm^3+^ content enhances pore volume and surface area, optimizing gas diffusion properties for sensing.

The adsorption–desorption isotherms of CZSmF are obtained in a N_2_ gas atmosphere at 300 °C (out gas temperature) for 60 min (out gas time) within the analysis time of 4.39 h. The obtained isotherms are displayed in Fig. 5 for the Zn compositions *x* = 0–0.04. The adsorbate (N_2_) volume increases gradually till 0.8 of the relative pressure (*P*/*P*_0_), and it increases rapidly after 0.8 of *P*/*P*_0_. The adsorption–desorption curves match well with type IV curves for the mesoporous materials.^32,33^

**Fig. 6:**
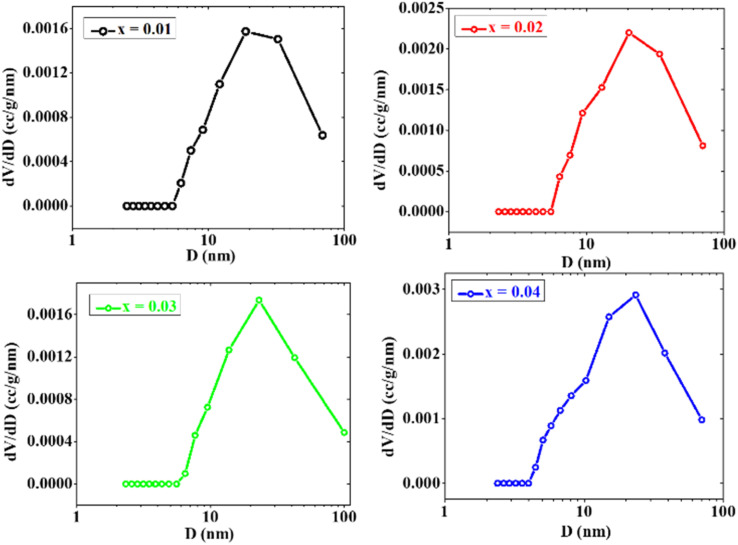
BJH pore size distribution curves for CZSmF ferrites (*x* = 0.01–0.04), demonstrating mesoporosity with peak pore sizes ranging between 20 and 30 nm. The *x* = 0.04 sample shows the highest pore volume and smallest average pore diameter, advantageous for enhancing gas adsorption kinetics.

The BJH pore size distribution curves displayed in Fig. 6 show the harmony in the variation for all the values of Sm doping in Co–Zn ferrites. The ferrites exhibit a diverse pore size distribution within the wide range of 3–70 nm. Such a wide range shows the possibility of the presence of a huge number of mesopores in each ferrite. The highest value of d*V*/d*D* is exhibited by the ferrite with *x* = 0.04 for a pore size of 20 nm having the highest pore volume of 0.124121 cc g^−1^. The ferrite with *x* = 0.01 exhibits the highest d*V*/d*D* peak at a pore size of 30 nm, attributed to the presence of relatively larger mesopores with an average size of 22.31 nm. This is associated with a smaller pore volume of 0.069291 cc g^−1^ compared to the other ferrite samples. The obtained values of surface area (*A*), total pore volume (*V*), and average pore radius (*D*) for all samples are listed in Table 1. The surface area varies from 6.2116 to 15.2336 m^2^ g^−1^, where the sample with a large surface area has a smaller pore size and bigger pore volume. The sample with a smaller (16.30 nm) pore size possesses a larger (0.124121 cc g^−1^) pore volume. In the case of spinel ferrites, it is reported that mesoporous samples with smaller average pore sizes exhibit higher porosity than the other materials.^33^

### 3.4 XPS analysis

**Fig. 7:**
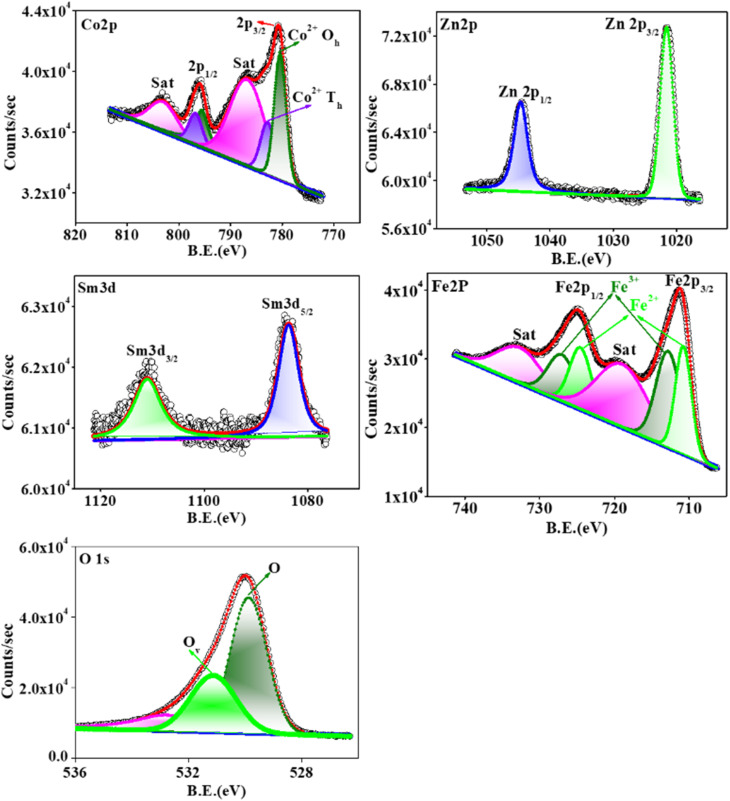
Deconvoluted XPS spectra of CZSmF01 (*x* = 0.01) showing binding energy peaks for Co 2p, Zn 2p, Fe 2p, Sm 3d, and O 1s. The data confirm valence states (Co^2+^, Zn^2+^, Fe^2+^/Fe^3+^, Sm^3+^) and reveal the presence of oxygen vacancies (peak at ∼531.13 eV in O 1s), which are critical for gas sensing behavior.

The oxidation states of the constituent elements of CZSmF are obtained from the analysis of the XPS spectra of a typical CZSmF sample with *x* = 0.01. The survey spectra of all the constituent elements at a glance are shown in Fig. S2 of SI 1. As observed from the figure, the spectra reveal the presence of Sm, Zn, Co, Fe, and O in the 5d, 2p, 2p, 2p, and 1s states. The spectrum of each element is deconvoluted to reveal the splitting of the states of each element, as shown in Fig. 7.

The binding energy (BE) values at the positions of the peaks are listed in Table S1 of the SI 1. A total of six peaks are visible in the deconvoluted spectra of Co. Out of these six peaks, peaks at binding energies 782.8 and 786.75 eV correspond to Co^2+^ ions in octahedral (O_h_) and tetrahedral (T_h_) sub-lattice sites^34^ respectively, while the satellite peaks at 786.75 and 803.05 eV confirm the presence of Co in the 2+ oxidation state.^35^ The deconvoluted spectra of Zn show two peaks at 1044.65 and 1021.54 eV, confirming the presence of Zn^2+^ cations.^36^ Two peaks at 1083.68 eV (3d_5/2_) and 1110.96 eV (3d_3/2_) in the deconvoluted spectra of Sm show the presence of Sm ions in the 3+ oxidation state.^37^ Deconvolution of two peaks in the Fe 2p spectrum, corresponding to 2p_3/2_ and 2p_1/2_ states, gives rise to six peaks. Out of which, the peaks at binding energies 710.77 and 724.5 eV show the presence of Fe ions in the 2+ oxidation state.^38,39^ The peaks at binding energies 712.79 and 727.04 eV show the presence of Fe ions in the 3+ oxidation state.^38,39^ The satellite peaks at 732.65 and 718.95 eV confirm the presence of Fe ions in the 2+ and 3+ oxidation states, respectively.^39^ The deconvolution of the O 1s spectrum gives rise to three different peaks, out of which the huge peak positioned at 529.86 eV corresponds to the O atoms within the CZSmF lattice.^40^ The middle one at 531.13 eV points towards the presence of oxygen vacancies^41^ within the lattice, and the one at 532.76 eV points to the presence of OH^42^ due to the absorption of H_2_O molecules by the CZSmF01 powder.

### 3.5 Analysis of gas sensing properties

**Fig. 8:**
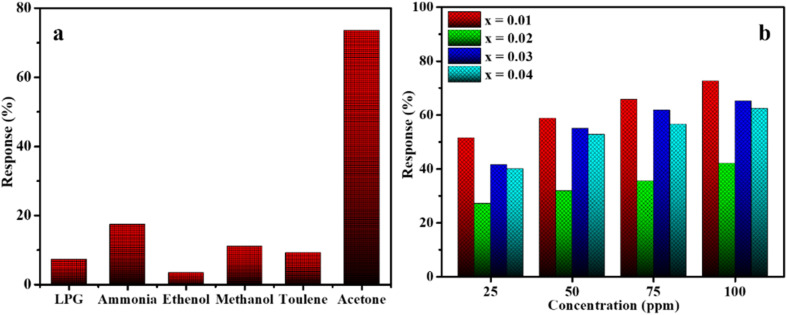
(a) Selectivity histogram of the CZSmF01 sensor response toward 100 ppm of different gases (LPG, NH_3_, EtOH, MeOH, toluene, and acetone) at room temperature, demonstrating high selectivity to acetone. (b) Gas response behavior of CZSmF ferrites (*x* = 0.01–0.04) toward varying acetone concentrations (25–100 ppm), highlighting the CZSmF01's relatively higher response efficiency.

The selectivity of the CZSmF gas sensors was analyzed against LPG, ammonia, ethanol, methanol, toluene and acetone gases. The selectivity experiment was performed for the typical CZSmF gas sensor with *x* = 0.01 (CZSmF01) at room temperature (296 K), and the results are displayed as a bar chart in Fig. 8a. A huge response is obtained for acetone gas, which is greater than 3.5 times the responses for the other gases in the experiment. Thus, the CZSmF gas sensor possesses better selectivity for acetone gas. The mesoporous structure of the CZSmF nanocrystals with a large surface area can adsorb a large amount of oxygen species. The conduction band electrons are trapped by these adsorbed oxygen molecules, increasing the potential barrier for electron transport and, consequently, raising the resistance of the CZSmF sensor surface. Upon exposure to acetone gas, the oxygen species on the surface of the sensor perform the oxidation of the gas, owing to which the electrons enter the CZSmF sensor, reducing its resistance. The two unpaired electrons of the acetone molecule enhance the reduction of resistance more effectively as compared to other gas molecules used in the experiment, which have no such free pairs of electrons. Due to such a good response to acetone gas, all compositions of CZSmF are tested for their responses to the gas, and the obtained results are displayed in Fig. 8b. As shown in the figure, the CZSmF01 sensor exhibits a relatively better response for all concentrations of acetone gas. BET analysis of CZSmF01 reveals a larger pore size accompanied by lower pore volume and surface area (Table 1). Such structural parameters, along with the high resistivity, are responsible for the relatively higher response recorded for the particular CZSmF01 sensor. In the case of spinel ferrite gas sensors, it is reported that the ferrites with larger pore size, pore volume, and surface area possess high sensitivity towards reducing gases.^13,43–45^ However, in the present CZSmF sensors, it is found that the CZSmF01 sensor, which has the second largest pore size, along with a smaller total pore volume and surface area possesses the highest response. This is attributed to the fact that, besides the surface area and pore volume, other factors affecting the gas sensing response are the pore size and electrical resistivity (discussed in SI 1).^46,47^ These two factors are better tuned in CZSmF01, which exhibits relatively higher values for both compared to the other CZSmF sensors. Thus, the pore size and electrical resistivity play an effective role in governing acetone gas sensing by the CZSmF nanocrystalline ferrites. Thus, it can be concluded that moderate pore size and high resistivity are crucial parameters deciding the response of a spinel ferrite gas sensor for reducing gases like acetone.

**Fig. 9:**
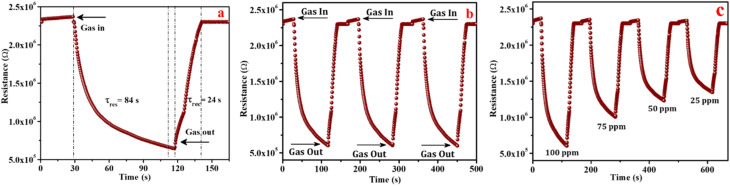
(a) Response and recovery transient profile of the CZSmF01 sensor for 100 ppm acetone gas at 296 K, showing n-type semiconductor behavior with response and recovery times of 84 s and 24 s respectively. (b) Reproducibility test over three cycles under identical conditions, confirming the sensor's stability and repeatability. (c) Dynamic transient responses at multiple acetone concentrations (25–100 ppm), validating the sensor's consistency and proportional sensitivity.

The gas sensing behavior, including the response and recovery of the CZSmF01 sensor, is displayed in Fig. 9a. It is a typical n-type semiconducting behavior of a metal oxide sensor wherein the resistance falls moderately after the entrance of gas in the chamber and rises rapidly on the exit of the gas.^48,49^ As shown in the figure, the initial resistance of the sensor in air (2.4 × 10^6^ Ω) decreases to 27% of this value (6.4 × 10^5^ Ω), when exposed to acetone gas. The time required for this much fall in resistance^50^ is recognized as the response time (*τ*_res_), which is 84 s for the present CZSmF01 sensor. This fall in resistance is recovered within the next 24 s after the exit of acetone gas from the chamber, which is known as the recovery time (*τ*_rec_). The literature reports that acetone gas sensor response times (*τ*_res_) and recovery times (*τ*_rec_) are as follows: Mn-substituted Ni ferrite sensors^51^ exhibit a *τ*_res_ of 180 s and *τ*_rec_ of 330 s, Ce-doped Mg ferrite sensors^52^ show a *τ*_res_ of 373.8 s and *τ*_rec_ of 393.6 s, and Zn_0.4_Fe_2.6_O_4_ sensors exhibit a *τ*_res_ of 4 s and *τ*_rec_ of 1108 s.

Generally, the gas response curve is divided into three parts *viz.*, a fast response curve immediately after the gas exposure, a slow response curve after a few seconds of gas exposure and a saturation curve. In the present case of CZSmF, the slow curve does not show saturation till the gas out time which indicates that chemisorption continues as long as the gas is in contact with CZSmF. The literature reports that such behavior in the gas response curve is due to the strong interaction between the active sites on the surface of the material with the gas molecules.^54^ In the present case, the active sites of the CZSmF surface strongly interact with the acetone molecule possessing a lone pair of free electrons owing to which the saturation does not occur till the gas out time.

The reproducibility of the CZSmF01 sensor is tested for 3 cycles at room temperature using 100 ppm acetone gas, and the results are shown in Fig. 9b. As observed from the reproducibility curves, the CZSmF01 sensor exhibits good reproducibility, showing consistent variations in resistance along with stable response and recovery times. Owing to its good reproducibility in sensing behavior, the sensor is further tested for its response to varying concentrations of acetone gas. The response of the sensor is depicted in Fig. 9c as dynamic transient curves for the 25–100 ppm concentration range at room temperature. The dynamic resistance of the sensor decreases with exposure to the gas from 25 ppm to 100 ppm, a behavior usually exhibited by n-type semiconducting metal oxide gas sensors.^13,55^

**Fig. 10:**
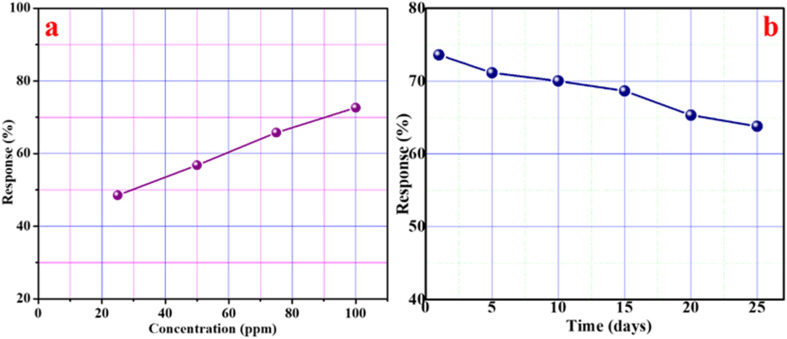
(a) Quantitative response analysis of CZSmF01 for acetone concentrations between 25 and 100 ppm, showing a linear sensor response indicating potential for concentration quantification. (b) Long-term stability study of CZSmF01 over 25 days under 100 ppm acetone exposure, confirming operational durability for practical applications.

The suitability of the sensor for the quantitative analysis of acetone gas is tested by recording the response for varying concentrations in the range of 25–100 ppm at room temperature. The obtained curve is displayed in Fig. 10a, which clearly shows that the response increases linearly with increasing concentration. Such behavior points toward the potential of CZSmF01 for the quantitative analysis of acetone gas.^12,53^ The long-term durability of the sensor is tested using 100 ppm acetone gas for 25 days at ambient temperature. The obtained curve (Fig. 10b) shows the stability of the sensor over a continuous period of 25 days.

#### 3.5.1 Gas sensing mechanism

The XPS analysis of CZSmF depicts the presence of oxygen vacancies within the ferrite crystal. The crystallization of the ferrites during sintering in the oxygen atmosphere gives rise to oxygen vacancies which induce the formation of Fe ions with mixed valence *i.e.*, Fe^2+^/Fe^3+^.^56^ In spinel ferrites, the oxygen vacancies are created at the octahedral sites.^57^ The role of oxygen vacancies in the gas-sensing mechanism is reported elsewhere.^58,59^ It is reported that the spinel ferrite, which is an n-type semiconductor in its stable state, reaches a metastable state due to the existence of oxygen vacancies, giving rise to excess electrons/holes in the conduction/valence band. Such a state of creation of oxygen vacancies can be represented by the chemical reaction^50^ as,5
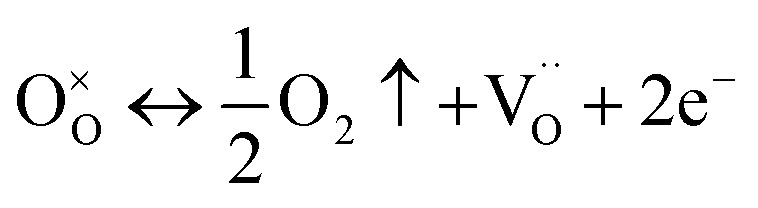
O^×^_O_, 
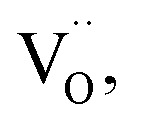
 O_2_, and e^−^ represent an unstable oxygen atom, oxygen vacancy at the oxygen lattice site, oxygen gas, and excess electrons (conduction band) respectively. The charge imbalance due to the removal of the unstable electron from the regular lattice site is balanced by the trapping of two excess electrons into the oxygen vacancy, and a donor level is created within the energy gap.^60–62^ Thus, along with the phenomenon of chemisorption, the oxygen from the environment interacts by its substitution within the vacancy. This substitution of environmental oxygen into the vacancy can be represented by the reversible reaction reported^51^ as,6
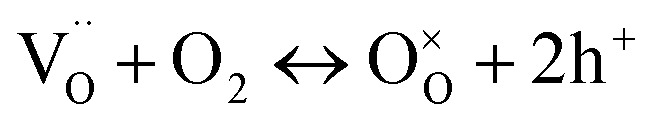
where h^+^ represents a free hole created due to the substitution of an oxygen ion (O^×^_O_) into an oxygen vacancy 
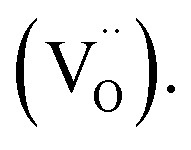
 Thus, the formation of oxygen vacancies contributes to the conductivity by increasing the concentration of holes.^63,64^

Chemisorption is the most favorable phenomenon reported in the case of spinel ferrites, where oxygen molecules upon contact with the surface of the ferrite transforms into negatively charged oxygen species, O_2(ads)_^−^, by the following reaction,

7 O_2(gas)_ → O_2(ads)_ + e^−^ → O_2(ads)_^−^

The O_2(ads)_^−^ captures free electrons from the conduction band of CZSmF and gets transformed into oxygen ions (O_(ads)_^−^) as given by,

8 O_2(ads)_^−^ + 2e^−^ → 2O_(ads)_^−^

Upon exposure of the CZSmF surface to acetone, the captured electrons are transferred back to the conduction band of the ferrite as represented by the reported^65^ reaction,

9 C_3_H_6_O_(gas)_ + 2O_(ads)_^−^ → CH_3_O^−^ + C^+^H_3_ + CO_2(gas)_ + 2e^−^

The transfer of captured electrons back to the sensor reduces its resistance. The two mechanisms, namely chemisorption (eqn (7)) and vacancy substitution (eqn (6)), dominate one another. Based on the dominance of the mechanism, the material works as an n/p-type semiconducting medium. n-type conduction took place in two steps: (i) adsorption of atmospheric oxygen resulting in an increase of resistance represented by eqn (7) and (8), and (ii) reduction of oxygen in the gas environment resulting in a decrease in resistance represented by eqn (9). In the present CZSmF01 sensor, this behavior is observed as shown in Fig. 9a. In the case of the ferrites, such n-type semiconducting behavior is most favorable owing to the hopping among the Fe cations possessing different valences (Fe^2+^ → Fe^3+^).^50,53,66^ The presence of Fe^2+^ and Fe^3+^ entities within the CZSMF01 sensor material is confirmed by XPS (Fig. 7) analysis. The presence of oxygen vacancies is also confirmed by XPS analysis, which is reported to facilitate the hopping of electrons by shifting the valence of Fe from 3+ to 2+.^57^ Our previous studies^12,13,49^ on gas sensing by rare earth doped Co–Zn ferrites have reported n-type semiconducting behavior. The facts of the present experimental study and the review of reported literature related to gas sensing by ferrites suggest the n-type semiconducting behavior of the present CZSmF01 sensor.

### 3.6 Effect of humidity

**Fig. 11:**
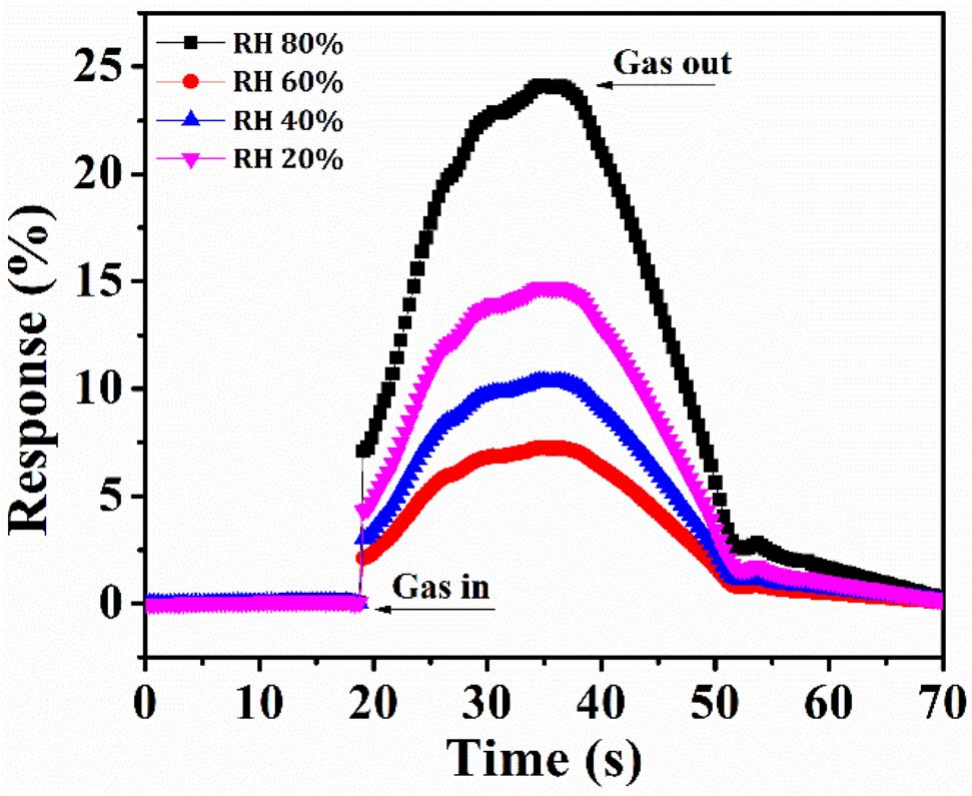
Relative humidity sensing profile of CZSmF01 in the 20–80% RH range at room temperature. Resistance variations indicate effective humidity detection with rapid response (10 s) and recovery (8 s) times, attributed to surface water adsorption and desorption dynamics.

The capability of the CZSmF01 sensor towards the sensing of humidity was studied by utilization of a binary electrode for the controlled levels of relative humidity varying from 20 to 80%. The relative humidity (RH) level at room temperature was controlled by using saturated salt solutions. The changing resistance of the CZSmF01 sensor with varying RH% is displayed in Fig. 11. The H_2_O molecules liberated by the salt solution behave as electron donors and reduce the sensor resistance with increasing RH. Due to such a mechanism of humidity sensing, the Fermi level of the material moves near the conduction band with the adsorption of H_2_O molecules on the surface of the sensor.^67^ The recorded values of response and recovery times for the CZSmF01 sensor are 10 s and 8 s respectively. The high surface area of the nanocrystalline CZSmF01 and the rapid desorption of the H_2_O molecules are responsible for the swifter response and recovery times. According to the reported literature,^68^ quick adsorption and desorption lower the process of thermodynamic adsorption of analyte molecules, resulting in easier desorption due to low adsorption energy. As a result, the response time is relatively longer than the recovery time.^68,69^ The enhanced performance of the sensor with varying RH conditions is depicted by the sensitivity of the CZSmF01 sensor, as shown in Fig. 11. In addition to its enhanced acetone sensing capabilities, CZSmF01 demonstrates effective humidity sensing with rapid response and recovery times. At room temperature, CZSmF01 achieved sensitivities of 75% for acetone and 24% for humidity, indicating its promise for commercial acetone gas sensing.

## 4 Conclusions

We report a novel Sm^3+^-doped Co–Zn ferrite (Co_0.7_Zn_0.3_Sm_*x*_Fe_2−*x*_O_4_) platform for highly selective, room-temperature acetone sensing, where performance is governed by the interplay of mesoporosity, pore size, and oxygen vacancies. Structural and surface analyses confirmed a defect-engineered spinel framework with tunable textural properties and significant vacancy concentrations, as directly validated by XPS.

The optimized composition, CZSmF01 (*x* = 0.01), uniquely combines moderate surface area, larger average pore size (∼22 nm), and high oxygen vacancy density to deliver fast response (84 s) and recovery (24 s), excellent selectivity, linear detection in the 25–100 ppm range, and long-term stability over 25 days. Its dual acetone–humidity sensitivity with rapid kinetics further expands its application potential.

This work provides the first direct correlation between mesopore architecture, electrical resistivity, and oxygen-vacancy-driven conduction in Sm^3+^-doped Co–Zn ferrites, establishing a clear structure–property–function relationship. The findings not only advance the understanding of rare-earth-modified ferrite sensing mechanisms but also present CZSmF01 as a robust, low-power candidate for next-generation ambient gas sensors.

## Conflicts of interest

There are no conflicts of interest to declare.

## Supplementary Material

NA-007-D5NA00631G-s001

## Data Availability

The data supporting this article have been included as part of the supplementary information (SI). See DOI: https://doi.org/10.1039/d5na00631g.
