# Supplementary material for: Room-temperature acetone gas sensing using Sm-doped Co–Zn ferrite nanoparticles: role of mesoporosity and oxygen vacancies in enhancing sensor response
Source: Nanoscale Adv. 2025 Sep 15;7(22):7259–72. doi: 10.1039/d5na00631g (PMC12495296; doi:10.1039/d5na00631g)
Supplement: NA-007-D5NA00631G-s001 [file NA-007-D5NA00631G-s001.pdf]

Supplementary Information (SI) for Nanoscale Advances.
This journal is © The Royal Society of Chemistry 2025

Supporting Information (SI)

(To be published as an electronic version only)

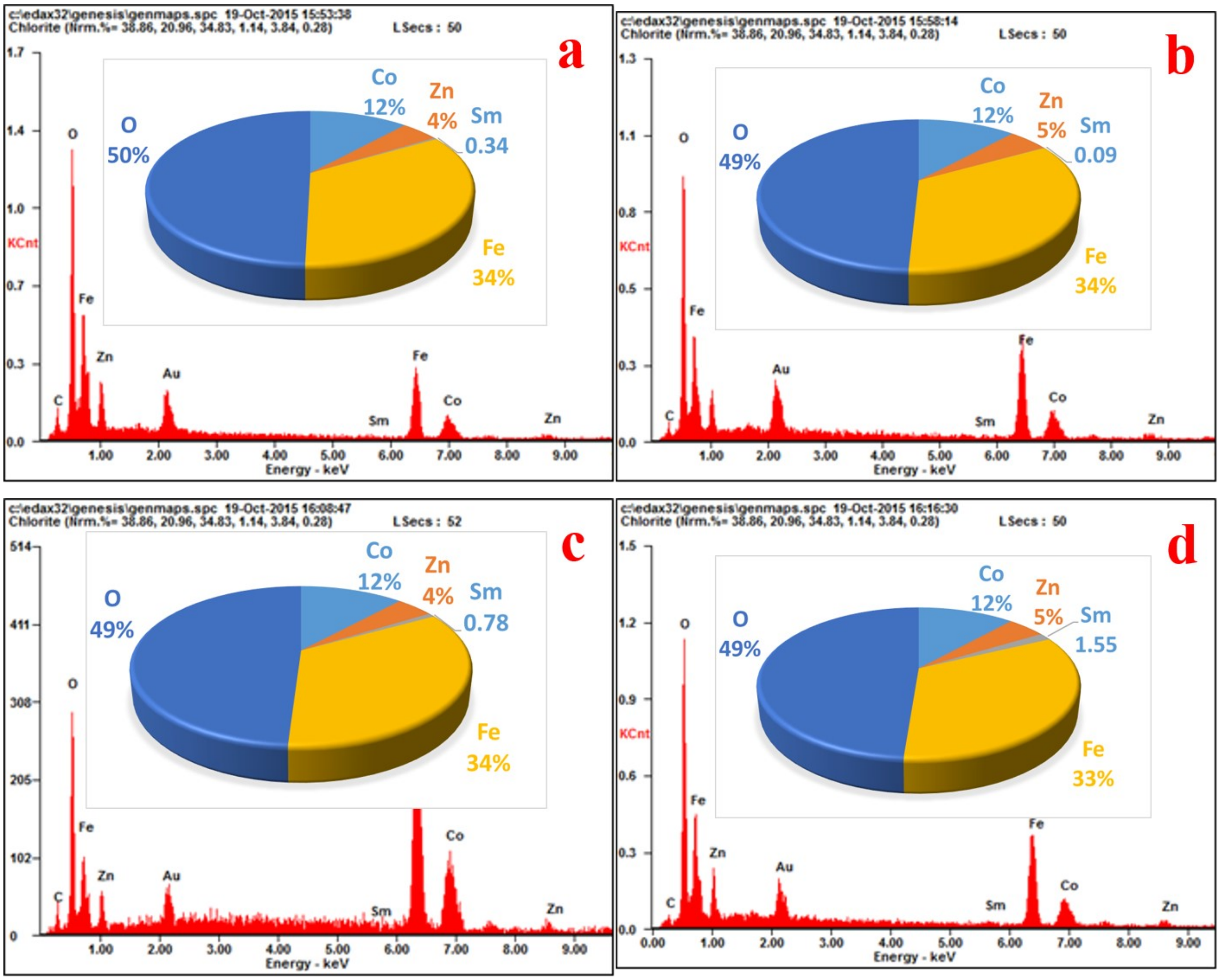

1. EDS spectra:

Fig. S1: The EDS spectra of CZSmF for x = 0.01(a), x = 0.02 (b), x = 0.03 (c), and x = 0.04 (d)

2. **XPS Spectra and binding energies:**

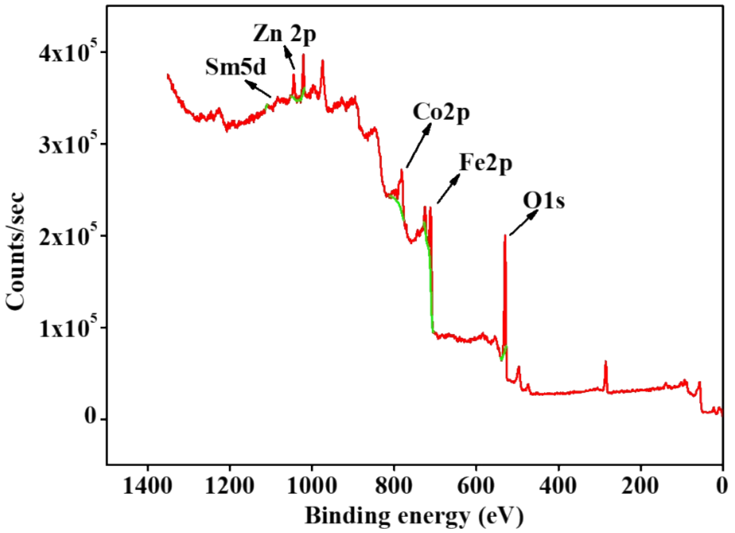

Fig. S2: The XPS survey spectrum of CZSmF with Zn compositions, x = 0.01.

Table S1: XPS data of the deconvoluted spectra of the elements in CZSmF.

Element	Position (eV)	Area	FWHM (eV)	GL%
Co 2p	803.049	13260.780	6.252	7
	795.549	10722.540	2.788	100
	796.763	6903.810	3.030	10
	786.749	47285.840	7.844	0
	782.800	11633.940	3.222	0
	780.401	23545.360	2.965	0
Fe 2p	732.645	38393.930	7.090	0
	727.042	31630.980	4.379	2
	724.542	33392.420	3.437	5
	718.948	77790.230	8.011	0
	712.786	57480.400	3.887	0
	710.774	41611.990	2.512	0
Zn 2p	1044.650	26030.590	2.557	59
	1021.543	37976.600	2.405	9
Sm3d	1083.675	11578.810	4.608	59
	1110.957	9291.323	6.271	84
O1s	532.763	19150.580	2.754	92
	529.875	62598.820	1.525	0
	531.129	32355.400	1.894	0

3. **Analysis of electrical conductivity:**

The frequency variation of a.c. electrical conductivity ($\sigma_{a.c.}$) is exhibited in Fig. 8. The magnitude of $\sigma_{a.c.}$ increases with frequency; initially gradually for low frequencies and later rapidly for high frequencies. The $\sigma_{a.c.}$ of ferrites depends upon the dielectric relaxation caused by the local displacement of the charge carriers. The electrical conduction in ferrites is due to the hopping of the electrons due to the exchange phenomena $Fe^{3+} \leftrightarrow Fe^{2+} + e^-$ that occurs at the octahedral B-site [S1]. This hopping of electrons increases with the increasing frequency of the electric field. Thus $\sigma_{a.c.}$ increased with frequency for high frequencies >10 KHz. However, for low frequencies up to 10 KHz the $\sigma_{a.c.}$ is almost constant independent of frequency; which may be because the hopping of electrons does not follow the frequency of the electric field. The compositional variation of $\sigma_{a.c.}$ for 1 MHz frequency is shown as an inset of Fig. 8. The $\sigma_{a.c.}$ is increasing with Sm composition (x). Samarium has 6 unpaired electrons in 4f orbital that contribute to the conduction in CZSmF and the conductivity increases. The increase of $\sigma_{a.c.}$ due to the coordination of 4f electrons of RE electrons is reported in the literature [S2]. Besides this, the oxygen vacancies which are detected by the XPS analysis of CZSmF play an important role in the increase of $\sigma_{a.c.}$. The oxygen vacancies in combination with the other lattice imperfections are reported to increase the electrical conductivity of the ferrite [S3]. In brief, the effects of Sm^{3+} substitution on electrical conductivity of the CZSmF can be summarized as follows.

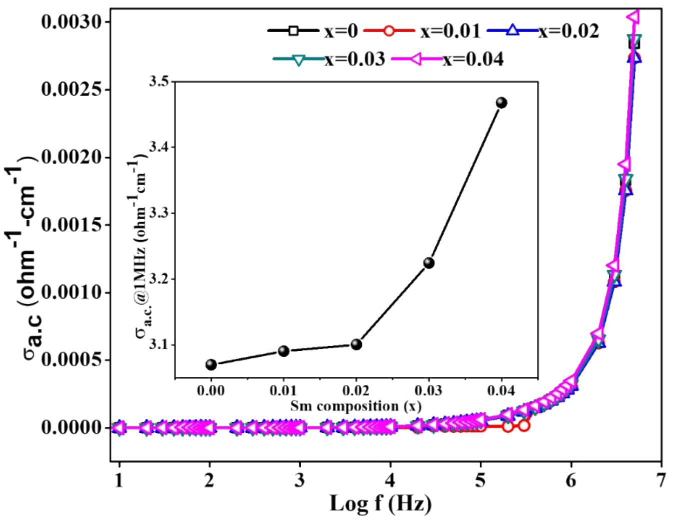

Fig. S3: The frequency and compositional variation of a.c. conductivity of CZSmF.

Owing to their ionic radius and +3 valence the substituted Sm^{3+} ions occupy the octahedral sites of the CZSmF lattice. The large Sm^{3+} (1.09 Å) ions replace the relatively smaller Fe^{3+} (0.64 Å) ions which gives rise to the lattice imperfections e.g., oxygen vacancies, due to the increased lattice strain. The

increasing composition of Sm^{3+} ions in the Co-Zn ferrite lattice increases the lattice strain and decreases the particle size (Table 1). The lattice imperfections and the fine particle size are creating interesting changes in the electrical conductivity of the CZSmF.

References:

[S1] R. Nongjai, S. Khan, K. Asokan, H. Ahmed, I. Khan Magnetic and electrical properties of In doped cobalt ferrite nanoparticles, J. Appl. Phys. 112 (2012) 084321.

[S2] S. Mahalaksmi, K.S. Manja, S. Nithiyanantham, Electrical properties of nanophase ferrites doped with rare earth ions, J. Supercond. Novel Magn. 27 (2014) 2083–2088.

[S3] S. Anjum, G.H. Jaffari, A.K. Rumaiz, M.S. Rafique, S.I. Shah, Role of vacancies in transport and magnetic properties of nickel ferrite thin films, J. Phys. D: Appl. Phys. 43 (2010) 265001.
